# Awareness, Beliefs, and Actions Concerning Zika Virus Among Pregnant Women and Community Members — U.S. Virgin Islands, November–December 2016

**DOI:** 10.15585/mmwr.mm6634a4

**Published:** 2017-09-01

**Authors:** Christine E. Prue, Joseph N. Roth, Amanda Garcia-Williams, Alison Yoos, Lena Camperlengo, Leah DeWilde, Mohammed Lamtahri, Andra Prosper, Cosme Harrison, Lauren Witbart, Irene Guendel, Douglas M. Wiegand, Natasha R. Lamens, Braeanna Hillman, Michelle S. Davis, Esther M. Ellis

**Affiliations:** ^1^Office of the Director, National Center for Emerging and Zoonotic Infectious Diseases, , CDC; ^2^Division of State and Local Readiness, Office of Public Health Preparedness and Response, CDC; ^3^Division of Violence Prevention, National Center for Injury Prevention and Control, CDC; ^4^Division of Global Health Protection, Center for Global Health, CDC;^5^Division of Reproductive Health, Center for Chronic Disease Prevention and Health Promotion, CDC; ^6^U.S. Virgin Islands Department of Health; ^7^Office of Public Health Scientific Services, Center for Surveillance, Epidemiology, and Laboratory Service, CDC; ^8^Office of the Director, Office of Public Health Preparedness and Response, CDC; ^9^Division of Surveillance, Hazard Evaluations, and Field Studies, National Institute for Occupational Safety and Health, CDC.

As of May 2, 2017, the U.S. Virgin Islands (USVI), comprising St. Thomas, St. John, and St. Croix, had reported 1,021 probable or confirmed cases* of Zika virus disease in its population of approximately 100,000 (*1*); 222 symptomatic and asymptomatic pregnant women in the USVI had tested positive for Zika virus. In January 2016, USVI Department of Health (USVI DOH) initiated Zika response measures, including surveillance, vector control, and a communications program. Interventions included education and outreach, distribution of Zika prevention kits^†^ to pregnant women in the USVI, and provision of free Zika virus laboratory testing and vector control services. In November 2016, USVI DOH staff members conducted interviews with convenience samples of community members and pregnant women to gather feedback about current and proposed interventions (*2*). Pregnant women reported taking a median of two actions to protect themselves from Zika, with repellent use being the most commonly reported action. Community members reported taking a median of one action and were supportive of several proposed vector control approaches. Whereas multiple pregnant women and community members reported hearing messages about the cause and consequences of Zika virus infections, few recalled messages about specific actions they could take to protect themselves. Integrating evaluation into response measures permits ongoing assessment of intervention effectiveness and supports improvement to serve the population’s needs.

During November 15–December 9, 2016, interviews were conducted with 269 pregnant women and community members living in the USVI to assess awareness, beliefs, and actions related to Zika virus and local prevention and control measures. USVI DOH interviewers identified commercial and clinic (public and private) locations to conduct interviews; these locations represented different locales and demographic groups of each island (*3*). Interviews were conducted in English and included open- and closed-ended questions. Pregnant women were asked about receipt, use, and usefulness of interventions including Zika prevention kits, laboratory testing, and vector control services. Community members were asked about their level of support for backpack spraying, spraying from trucks, spraying from airplanes, and placement of mosquito traps in yards. Interviewers received training on obtaining consent for participation and use of the interview instruments and Epi Info for Mobile Devices,^§^ which permitted audio recording of questions and responses. This project was determined by CDC as not subject to Institutional Review Board review. An Atlanta-based analytics team reviewed audio files and provided feedback to field staff members to improve fidelity to protocols, analyzed closed-ended and multiple-choice responses, and transcribed, coded, and analyzed responses to open-ended questions.

A systematic process for tallying the number of interview requests and refusals was not used; however, refusals were rare. The final sample included 269 completed interviews with 104 (38.7%) pregnant women and 165 (61.3%) nonpregnant community members, including 120 (44.6%) participants on St. Croix, 116 (41.3%) on St. Thomas, and 33 (12.3%) on St. John (Table 1). The median age of pregnant women respondents was 27 years (range = 18–43 years). Among 95 pregnant respondents for whom information on race/ethnicity and education was available, 58 (61.1%) were non-Hispanic black, 28 (29.5%) were Hispanic, and eight (8.4%) were non-Hispanic white. Thirty-six (37.9%) pregnant respondents were high school graduates, 25 (26.3%) attended some college, 17 (17.9%) were college graduates, and six (6.3%) had postgraduate education. Most pregnant women were in their third (48.1%) or second (37.5%) trimester of pregnancy.

**TABLE 1:** Demographic characteristics of pregnant women and community member respondents — U.S. Virgin Islands Department of Health, U.S. Virgin Islands, November–December 2016

Characteristic	No. (%)	
	Pregnant women (N = 104)	Community members (N = 165)
**Location of interview**		
**St. Croix**	**51 (49.0)**	**69 (41.8)**
**St. Thomas**	**45 (43.3)**	**71 (43.0)**
**St. John**	**8 (7.7)**	**25 (15.2)**
Sex*		
Male	NA	74 (45.7)
Female	104 (100)	88 (54.3)
**Age group (yrs)**		
18–24	38 (36.5)	11 (7.1)
25–34	42 (40.4)	34 (21.8)
35–44	13 (12.5)	32 (20.5)
**45–54**	**0 (—)**	**38 (24.4)**
**55–64**	**0 (—)**	**29 (18.6)**
**≥65 **	** 0 (—)**	** 12 (7.7)**
**Refused**	**11 (10.6)**	**0 (—)**
Highest level of education*		
**None**	**1 (1.1)**	**0 (—)**
Preschool through grade 12	10 (10.5)	51 (31.5)
High school diploma	36 (37.9)	43 (26.5)
Some college	25 (26.3)	45 (27.8)
College graduate	17 (17.9)	11 (6.8)
Postgraduate	6 (6.3)	12 (7.4)
Missing	9 (8.7)	0 (—)
**Race/Ethnicity^†^**		
White, non-Hispanic	8 (8.4)	17 (10.6)
Black, non-Hispanic	58 (61.1)	113 (70.2)
Hispanic	28 (29.5)	21 (13.0)
Other, non-Hispanic	1 (1.0)	1 (0.6)
Refused	0 (—)	9 (5.7)
**Pregnancy trimester**		
First	14 (13.5)	NA
Second	39 (37.5)	NA
Third	50 (48.1)	NA
Missing	1 (1.0)	NA

**Abbreviation:** NA = not applicable.

* Proportion for community members is from a sample of 162 community members.

^†^ Proportion for pregnant women is from a sample of 95; proportion for community members is from a sample of 161.

Among 165 community members who were interviewed, 74 (45.7%) were male; the median age was 45 years (range = 18–81 years); 113 (70.2%) were non-Hispanic black, 21 (13.0%) were Hispanic, and 17 (10.6%) were non-Hispanic white. Fifty-one (31.5%) had less than a high school education, 43 (26.5%) were high school graduates, 45 (27.8%) had attended some college, 11 (6.8%) were college graduates, and 12 (7.7%) had postgraduate education.

Pregnant women provided a median of two responses (range = 1–5) to the question, “What have you heard about Zika?” and the most common responses were that Zika causes microcephaly or brain defects in babies (67.3%) and is transmitted by mosquitoes (34.6%) (Table 2). Community members provided a median of one response (range = 0–5); the most common response was that Zika is transmitted by mosquitoes (48.5%). Only 11.5% of pregnant women and 9.1% of community members reported hearing that Zika virus can be sexually transmitted. Less than 3% of pregnant women or community members mentioned hearing about individual actions that could be taken to prevent Zika virus infection. Only 3.8% of pregnant women and 6.1% of community members stated that Zika virus transmission was occurring in the USVI.

Among 103 pregnant women, 56 (54.4%) reported being moderately or extremely concerned about becoming infected with Zika virus. Whereas 14 (13.9%) of 101 pregnant women stated it was likely or extremely likely that they would become infected, 86 (83.5%) of 103 said they were confident or very confident in their ability to protect themselves and their baby from infection during their pregnancy. Zika virus was reported as a serious or very serious health concern to the community by 124 (75.6%) community members, and to them personally by 82 (49.7%), with 69 (41.8%) stating that it was likely or very likely that they would become infected (Table 2). A majority of pregnant women and community members reported having either no conversations or only one or two conversations with family members or friends about Zika in the past month (Table 2).

**TABLE 2:** Awareness, risk perceptions, and actions taken among pregnant women and community members — U.S. Virgin Islands (USVI) Department of Health, U.S. Virgin Islands, November–December 2016

Awareness of Zika*	No. (%)	
	Pregnant women (N = 104)	Community members (N = 165)
**Health effects of Zika**		
Causes microcephaly or brain defects in babies	70 (67.3)	53 (32.1)
Pregnant women should try not to get it	23 (22.1)	12 (7.3)
Causes fever, rash, and conjunctivitis	14 (13.5)	19 (11.5)
Dangerous	4 (3.8)	12 (7.3)
Like dengue and chikungunya	3 (2.9)	12 (7.3)
Can be life-threatening can cause paralysis, GBS	1 (1.0)	1 (0.6)
**Transmission**		
Can get it from mosquitoes	36 (34.6)	80 (48.5)
Can be transmitted by sex from a man to a woman	12 (11.5)	15 (9.1)
Persons in USVI are getting infected with Zika	4 (3.8)	10 (6.1)
**Protective Actions **		
Wear repellent	3 (2.9)	1 (0.6)
Wear clothing that covers arms and legs	1 (1.0)	0 (—)
Eliminate standing water	2 (1.9)	3 (1.8)
Put screens on windows and doors	1 (1.0)	0 (—)
**Haven’t heard anything**	2 (1.9)	16 (9.7)
**Other (specify)** ^†^	17 (16.3)	55 (33.3)
**Beliefs about risk**		
Serious or very serious health concern to you personally	NA	82 (49.7)
Serious or very serious health concern to your community	NA	124 (75.6)
Moderately or extremely concerned about Zika virus for yourself and your baby^§^	56 (54.4)	NA
Likely or extremely likely that you will be infected with the Zika virus (for pregnant women: infected during your pregnancy)^¶^	14 (13.9)	69 (41.8)
Confident or very confident in your ability to protect yourself from getting infected with the Zika virus during your pregnancy^§^	86 (83.5)	NA
Personal protective behaviors**		
Used repellent	77 (74.0)	70 (42.4)
Wore clothes that cover arms and legs	28 (26.9)	13 (7.9)
Sprayed permethrin on clothes	11 (10.6)	0 (—)
Used mosquito net at night	11 (10.6)	4 (2.4)
Don’t go outside at all	10 (9.6)	2 (1.2)
Used mosquito net during the day	6 (5.8)	1 (0.6)
Used a condom/had my partner use a condom in all sexual relations	6 (5.8)	1 (0.6)
Don’t go outside at night	3 (2.9)	4 (2.4)
Got tested and/or got my partner tested for Zika	1 (1.0)	0 (—)
Abstained from sexual intercourse	0 (—)	2 (1.2)
Prayed to God	0 (—)	2 (1.2)
Looked for more information about Zika	0 (—)	1 (0.6)
**Mosquito control around home**		
Removed standing water	18 (17.3)	50 (30.3)
Sprayed inside my home	14 (13.5)	24 (14.6)
Put screens on windows and doors	14 (13.5)	17 (10.3)
Sprayed outside my home	11 (10.6)	8 (4.8)
Used mosquito coil/light fires to keep mosquitoes away	10 (9.6)	14 (8.5)
Closed windows and doors	8 (7.7)	12 (7.3)
Used larvicides (like mosquito dunks)	4 (3.8)	4 (2.4)
Cleaned household environment	4 (3.8)	5 (3.0)
Used air conditioning	3 (2.9)	2 (1.2)
Cleaned/Scrubbed water source/storage unit/water container(s)	1 (1.0)	3 (1.8)
Cut grass	1 (1.0)	6 (3.6)
Put cover(s) over the water source/storage unit/water container(s)	0 (—)	6 (3.6)
**Haven’t done anything**	3 (2.9)	26 (15.8)
**No answer given**	8 (7.7)	3 (1.8)
**Other (specify)** ^††^	25 (24.0)	32 (19.4)
**Frequency of conversation with family members and friends about Zika in past month^†^**		
Not at all	25 (24.3)	63 (38.2)
Only once or twice	33 (32.0)	45 (27.3)
Sometimes	24 (23.3)	26 (15.8)
Often	15 (14.6)	22 (13.3)
Every day	6 (5.8)	9 (5.5)

**Abbreviations:** GBS = Guillain-Barré syndrome; NA= not applicable.

* Responses given to the open-ended question, “What have you heard about Zika?”

^†^ Pregnant women: a lot of things, how to prevent, how it’s spread, and large outbreaks in Brazil. Community members: affects pregnant women, makes them sick, it’s a virus, it came from other countries, Zika is bad and should be avoided, theories on how it got here, it’s like the flu, tires and drums a source.

^§^ Proportion is from a sample of 103 pregnant women.

^¶^ Proportion is from a sample of 101 pregnant women.

** Responses to open-ended questions. Pregnant women were asked**, “**What actions have you taken to protect yourself from getting infected with the Zika virus since you found out you were pregnant?” Community members were asked, “What actions have you taken to protect yourself from getting infected with the Zika virus?”

^††^ Pregnant women: used Zika prevention kit, avoided areas with mosquitoes, wore no perfume, and protection used by family. Community members: used herbal remedies, used a mosquito swatter, sprayed in general (no place specified), avoid mosquitoes, maintained a healthy lifestyle, kept mosquitoes out of house, and wore light colored clothing.

When asked, “What actions have you taken to protect yourself from getting infected with Zika virus since you found out you were pregnant?” women reported taking a median of two actions (range = 0–6) with use of mosquito repellent (74.0%) and wearing clothing that covers arms and legs (26.9%) as the most frequently reported actions (Table 2). When community members were asked what actions they had taken to protect themselves, they reported taking a median of one action (range = 0–9) with use of mosquito repellent (42.4%) the most commonly reported action (Table 2).

Pregnant women were asked questions about their receipt of specific interventions and performance of specific behaviors. Among 97 pregnant women, 69 (71.1%) reported having received a Zika prevention kit (Table 3) with 67.2% stating that the repellent was the most important item in the kit and the one most frequently depleted. Among 95 pregnant women for whom information on Zika testing was available, 74 (77.9%) reported having been tested; 67.6% reported receiving their test results within 2 weeks; 22 (22.4%) reported that their partner had also been tested. Among 97 pregnant women, 48 (49.5%) said they heard about the availability of vector control services. Among the 31 pregnant women who reported hearing about and being offered vector control services, 25 wanted the service and 21 had been contacted by the USVI DOH to schedule the appointment for service delivery. Twenty (80%) of the 25 pregnant women who wanted vector control services reported receiving them.

**TABLE 3:** Receipt of interventions and self-reported performance of recommended Zika prevention behaviors among pregnant women — U.S. Virgin Islands Department of Health, U.S. Virgin Islands, November–December 2016

Interventions/Behaviors	No. (%) Pregnant women (N = 104)
**Interventions received**	
Zika Prevention Kit*	69 (71.1)
**Pregnant woman test for Zika virus^†^**	**74 (77.9)**
**Husband/Partner test for Zika virus^§^**	**22 (22.4)**
**Vector control services around home (among 25 respondents who desired services)**	**20 (80.0)**
Responded affirmatively to closed-ended questions about recommended Zika prevention behaviors	
**Used mosquito repellent yesterday^¶^**	**44 (43.1)**
**Used a condom every time they had sex (among 81 sexually active respondents)**	**15 (18.8)**
**Never used a condom when they had sex (among 81 sexually active respondents)**	**46 (57.5)**
**Removed standing water^†^**	**27 (28.4)**
**Used mosquito bed net yesterday^¶^**	**13 (12.7)**
**Interviewer observation**	
Wearing long pants right now**	45 (45.5)
Wearing long sleeved shirt right now**	22 (22.2)

* Among a sample of 97 pregnant women.

^†^ Among a sample of 95 pregnant women.

^§^ Among a sample of 98 pregnant women.

^¶^ Among a sample of 102 pregnant women.

**Among a sample of 99 pregnant women.

Among 102 pregnant women, 44 (43.1%) reported using insect repellent in the last 24 hours, 13 (12.7%) reported having slept under a bed net in the last 24 hours, and 27 (28.4%) reported removing standing water from their property in the past week (Table 3). Among 81 pregnant women who reported having sexual intercourse since becoming pregnant, only 15 (18.8%) reported using a condom every time they had sex, whereas 46 (57.5%) reported they never used a condom. At the time of the interview, 45.5% of pregnant women were observed to be wearing long pants and 22.2% were wearing long sleeves.

Community members were asked about their level of support for vector control methods to reduce mosquito populations in their community. Among those who responded, most supported or strongly supported putting mosquito traps in their yard (91.2%) and backpack spraying (75%); 66.5% and 23.9% supported spraying from trucks and airplanes, respectively.

## Discussion

Most USVI respondents believed that Zika is a serious health concern. Reported levels of concern about Zika virus infection among USVI respondents were slightly higher than those reported in surveys conducted in the continental United States (*4*–*8*). Two thirds of pregnant women and one third of community members in the USVI mentioned that Zika virus infection can cause serious birth defects. Surveys of residents of the continental United States showed wide variation in knowledge of the link between Zika virus infection and birth defects (*4*,*6*,*7*,*9*), with USVI residents generally having slightly higher knowledge levels than did respondents to the U.S.-based surveys. Approximately one third of pregnant women and less than half of community members in USVI mentioned that Zika virus can be transmitted by mosquitos and few pregnant women and community members (11.5% and 9.1%, respectively) mentioned that Zika can be sexually transmitted, suggesting gaps in awareness of modes of transmission, and possibly, reluctance to discuss sex. U.S. surveys revealed a similar pattern, with most respondents aware that Zika is spread by mosquitoes and fewer respondents aware of sexual transmission (*5*–*10*). Although the majority of pregnant respondents expressed concern about Zika, they also reported a high level of confidence in their ability to protect themselves and their baby and a belief that it was unlikely that they would become infected during their pregnancy, despite the relatively low reported prevalence of practicing protective behaviors. Reasons for this incongruity are not clear. It is possible that pregnant women were unaware of local cases of Zika virus disease, because <4% of them mentioned Zika virus transmission in USVI, or that Zika prevention messages were not reaching pregnant women, because <3% mentioned hearing messages about protective actions. That pregnant women reported limited recent conversations with family or friends about Zika might indicate a relative lack of importance of Zika in their lives or desensitization associated with living in an area where vectorborne diseases are prevalent. In addition, most pregnant participants were in their second or third trimester, and might have believed that protective actions were less essential later in pregnancy. Finally, women’s confidence might have been related to their receiving Zika-related services and to beliefs in their effectiveness.

Information collected from this assessment enabled program planners to tailor activities to address needs. For example, the community support of traps, backpack spraying, and truck spraying were important in developing USVI’s vector control plan. This assessment also provided feedback to USVI DOH staff members about how messages were being received, perceived, and acted upon. Recognizing prevention program strengths and deficiencies allowed program planners to reframe and refocus messaging to educate the public about transmission and emphasize protective actions.

The findings in this report are subject to at least three limitations. First, the convenience sample of interview venues were selected by USVI DOH staff members most familiar with their communities to ensure demographic diversity and were not population-based. Second, because the interviews were conducted during the Zika response, answers from respondents might have been subject to social desirability bias. Finally, responses were self-reported and not verified. 

Despite these limitations, gaining insights into the awareness, beliefs, and actions of USVI pregnant women and community members allowed Zika responders to improve messaging and bolster the response effort. Building in a rapid assessment during an outbreak response offers an essential feedback loop to local public health authorities about their interventions.

**Summary**

**What is already known about this topic?**

U.S.-based surveys conducted throughout 2016 have shown high levels of awareness of the Zika virus outbreak, moderate levels of concern about Zika, and low levels of knowledge about how Zika is transmitted.

**What is added by this report?**

Zika-related awareness, beliefs, and actions among residents of the U.S. Virgin Islands, who are not included in U.S.-based surveys, were assessed in interviews of pregnant women and community members. Multiple respondents reported hearing that Zika virus is transmitted by mosquitoes and causes microcephaly in babies. Fewer mentioned hearing about sexual transmission of Zika virus or what actions to take to prevent infection. Most respondents reported Zika virus as a serious concern although there were varying levels in perceptions of susceptibility and protective actions taken. Most pregnant women reported receiving interventions offered to them and most community members expressed support for several vector control approaches.

**What are implications for public health practice?**

The feedback from these interviews helped the U.S. Virgin Islands Department of Health identify information gaps that can be addressed through communication, education, and community engagement. Gathering feedback about key aspects of a response effort from community members is vital to ensure that interventions reach them and are translated into effective prevention programs.

## Footnotes

* These cases include immunoglobulin M–probable and polymerase chain reaction/plaque-reduction neutralization testing confirmed cases of Zika virus disease among symptomatic persons; asymptomatic pregnant women are not included.

† The Zika prevention kit included the following items: insect repellent, permethrin, condoms, a mosquito bed net, mosquito dunks (a larvicide used to treat standing water), and educational materials.

§ https://www.cdc.gov/epiinfo/mobile.html.
